# 500 bee stings in a single fatal encounter

**DOI:** 10.1177/00258172241241463

**Published:** 2024-06-13

**Authors:** Tanya Singh, Jagadish R Padubidri, Akshath KS, Sowmya J Rao, Matthew A Manoj

**Affiliations:** 1 Kasturba Medical College, Mangalore, Manipal Academy of Higher Education, India; 2 Department of Forensic Medicine and Toxicology, Kasturba Medical College Mangalore, Manipal Academy of Higher Education, Manipal, India; 3 Department of Paediatrics, SNR District Hospital, Kolar, India; 4 Department of Oral Pathology, Sharavathi Dental College, Shimoga, India; 5 Kasturba Medical College, Mangalore, Manipal Academy of Higher Education, India

**Keywords:** Bee sting, apiphobia, anaphylaxis, multi-organ, sudden death

## Abstract

Incidences of multiple bee stings have been increasing globally; the substantial amount of bee venom injected in such incidents can result in anaphylactic shock, rhabdomyolysis and renal failure, proving fatal in some cases. While anaphylactic reactions are more common and have established treatment protocols, inflammatory response induced by the venom demands a tailored approach. Here we report a case of a 70-year-old male in India who succumbed from approximately 500 bee stings. Based on our literature review, this case stands out as one of the first reported fatalities caused by 500 bee stings in our country. The unidentified species of bees in this case makes management of such bee venom-related toxic reactions more difficult. This report emphasises the importance of prompt and appropriate interventions.

## Introduction

Honeybees, belonging to the Apis species, are social insects; they live in communities and play a significant role as pollinators in agriculture. Bee stings are common in rural areas, among farmers and agriculturists. They commonly cause local hypersensitivity reactions like pain, swelling and redness. However, multiple stings can prompt systemic reactions such as anaphylaxis, resulting in sudden death.^1^

It has been reported that insect bites are responsible for anaphylaxis in approximately 3% of the world’s population.^2^ According to data published by the Centers for Disease Control and Prevention, a total of 788 deaths occurred from hornet, wasp and bee stings during 2011–2021 in the United States.^3^ It is estimated that approximately 500 stings are required to cause death by direct toxicity, but fewer stings can be fatal in children.^4^

Several deaths have also been reported to occur as a result of massive poisoning due to the toxin effects of the bee stings without any allergic manifestations. Some of the rarer complications associated with multiple stings include rhabdomyolysis, myocardial infarction, acute kidney injury, thrombocytopenia, hepatic injury and shock.^5^

## Case details

A 70-year-old farmer had gone to the field in the afternoon, where he was attacked by a swarm of bees. He was brought to a primary health centre and then shifted to the district hospital for management, where he was declared dead upon arrival.

**Figure 1.**
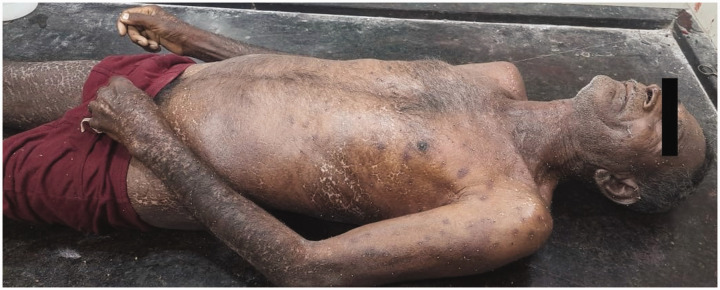
Multiple bee stings present all over the body.

**Figure 2.**
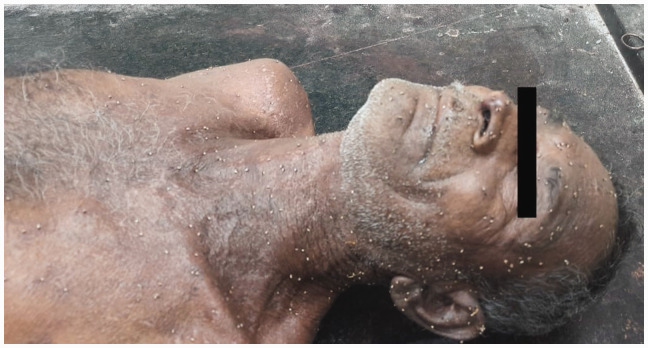
Multiple bee stings present on the face and neck.

**Figure 3.**
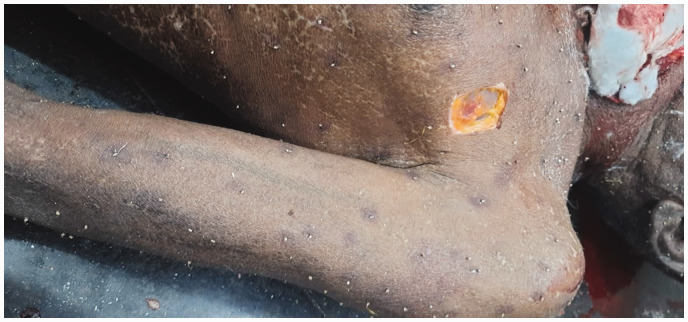
Multiple bee stings present in the thorax and upper limb.

**Figure 4.**
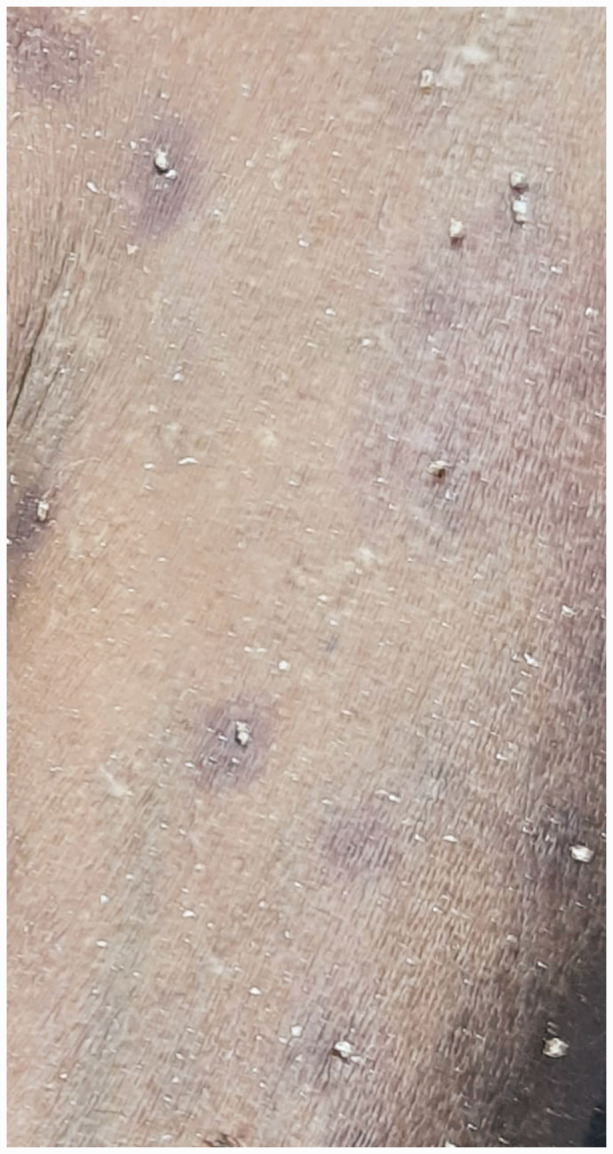
Stingers present in situ at the sites of bee stings.

Autopsy revealed approximately 500 bee stings (Figure 1). Stings were present all over the body, more concentrated on the face (Figure 2), upper limbs, thorax (Figure 3 and 4) and abdomen, with stingers in situ (Figure 4). There was presence of rigor mortis and fixed post-mortem lividity over the back. On internal examination, brain and spinal cord were found intact. Examination of mouth, pharynx and oesophagus showed swollen tongue and congested mucosa. Larynx and trachea were oedematous and congested. Pleural membranes were intact. Cut sections of both lungs showed congestion. Large vessels were patent; pericardium and heart were unremarkable. Stomach lining showed congestion; intestinal mucosa was congested along with presence of faecal matter in the large intestine. Liver, spleen and pancreas were unremarkable. Cut sections of both kidneys showed congestion. Urinary bladder and genitalia were also intact. The cause of death was opined as death due to laryngeal oedema secondary to anaphylactic shock following multiple bee stings.

## Discussion

Hymenoptera, which refers to the membranous wings of the insects, is one of the largest insect orders, comprising wasps, bees and ants. Hymenoptera antigen causes severe delayed reaction such as “serum sickness, neurological disturbances, acute renal failure, haemolysis, thrombotic thrombocytopenic purpura (TTP), disseminated intravascular coagulation (DIC), and cardiac arrhythmias”.^6^ Atypical reactions can occur hours or days after the stings.^6^ Hypersensitivity from bee stings commonly presents as mild urticaria or angioedema and in certain cases can also result in anaphylaxis. A rare presentation of multi-organ failure can occur due to massive envenomation caused by multiple bee stings, ranging from 50 to more than 500 stings.^5^ The severity of systemic toxicity caused by the envenomation is dependent on the individual’s age, body weight, immune response, sensitisation, etc.^7^

Research has shown that bee venom consists of various enzymes and toxins such as melittin, apamin, phospholipase A_2_, histamine and hyaluronidase. Melittin, the primary component, causes inflammation, intravascular haemolysis, and rhabdomyolysis, which leads to acute kidney injury. Histamine receptors affect the cardiac tissue causing myocardial injury. Cardiac involvement although rare is the most significant life-threatening complication.^2^ In a study by Chapsa et al., six significant risk factors were identified for anaphylaxis, which include “interval from sting to reaction, absence of urticaria or angioedema during anaphylaxis, older age, male sex, elevation of baseline serum tryptase (BST) level, and diagnosis of systemic mastocytosis”. But no association was established between severe anaphylaxis and comorbidities and medications.^8^

Allergic and systemic toxic reactions are difficult to differentiate initially; however, the treatment remains the same for both in the initial few minutes. The specific treatment against bee envenomation would be an antivenom therapy, but as of now no such antivenom is available. Emergency management and clinical monitoring of patients plays a crucial role in such circumstances.^7^

With rising apiculture industry worldwide for honey production, along with changes in the natural habitat of honeybees due to climate change, the incidence of bee stings is on the rise. Currently, limited literature is available and the further limitations of a proper reporting system pose a challenge in accurately estimating the prevalence of such incidents. Although commonly found honeybee species in India include *Apis dorsata*, *Apis florea*, *Apis cerana indica* and *Apis mellifera*, we could not find data regarding the natural habitats and behaviour of these species in the literature.^9^ Identifying the particular species of bees poses an additional challenge in most cases. This is a unique case as it is one of the very few reported deaths due to multiple bee stings. This case highlights the importance of a proper documentation and reporting system particularly in developing countries where farming and apiculture are crucial economic resources.

## Conclusion

Multiple bee stings led to massive envenomation causing multi-organ failure and sudden death secondary to anaphylaxis. Therefore, it is essential for patients to receive prompt and appropriate management. Monitoring is essential to avoid fatalities. Improved research clinical trials are needed to get a better understanding of the pathogenesis of the various types of bee toxins and to aid in providing appropriate treatment modalities.
